# Herpes Virus MicroRNA Expression and Significance in Serous Ovarian Cancer

**DOI:** 10.1371/journal.pone.0114750

**Published:** 2014-12-08

**Authors:** Deep Pandya, Marisa Mariani, Mark McHugh, Mirko Andreoli, Steven Sieber, Shiquan He, Candice Dowell-Martino, Paul Fiedler, Giovanni Scambia, Cristiano Ferlini

**Affiliations:** 1 Danbury Hospital Research Institute, Danbury, CT, United States of America; 2 Department of Gynecology, Catholic University of the Sacred Heart, Rome, Italy; Gustave Roussy, France

## Abstract

Serous ovarian cancer (SEOC) is the deadliest gynecologic malignancy. MicroRNAs (miRNAs) are a class of small noncoding RNAs which regulate gene expression and protein translation. MiRNAs are also encoded by viruses with the intent of regulating their own genes and those of the infected cells. This is the first study assessing viral miRNAs in SEOC. MiRNAs sequencing data from 487 SEOC patients were downloaded from the TCGA website and analyzed through in-house sequencing pipeline. To cross-validate TCGA analysis, we measured the expression of miR-H25 by quantitative immunofluorescence in an additional cohort of 161 SEOC patients. Gene, miRNA expression, and cytotoxicity assay were performed on multiple ovarian cancer cell lines transfected with miR-H25 and miR-BART7. Outcome analysis was performed using multivariate Cox and Kaplan-Meier method. Viral miRNAs are more expressed in SEOC than in normal tissues. Moreover, Herpetic viral miRNAs (miR-BART7 from EBV and miR-H25 from HSV-2) are significant and predictive biomarkers of outcome in multivariate Cox analysis. MiR-BART7 correlates with resistance to first line chemotherapy and early death, whereas miR-H25 appears to impart a protective effect and long term survival. Integrated analysis of gene and viral miRNAs expression suggests that miR-BART7 induces directly cisplatin-resistance, while miR-H25 alters RNA processing and affects the expression of noxious human miRNAs such as miR-143. This is the first investigation linking viral miRNA expression to ovarian cancer outcome. Viral miRNAs can be useful to develop biomarkers for early diagnosis and as a potential therapeutic tool to reduce SEOC lethality.

## Introduction

Serous ovarian cancer (SEOC) is the most lethal gynecologic malignancy. Due to its clinical indolence, the majority of patients are diagnosed late stage when surgery alone is insufficient to completely eradicate the tumor. As a consequence, chemotherapy is usually required to further control the disease. First-line chemotherapy for ovarian cancer typically includes a platinum agent (usually carboplatin) and a taxane (usually paclitaxel) [1]. Biomarkers which are prospectively predictive of sensitivity or resistance to chemotherapy are desperately needed to properly individualize therapeutic options and avoid toxic treatments for those patients who will be refractory to chemotherapy. The task of developing such biomarkers, problematic for all solid malignancies, is particularly vexing for ovarian cancer wherein extreme clonal heterogeneity is the norm and for which no driving mutations have been identified [2].

MicroRNAs (miRNAs) are a class of small, noncoding RNAs which regulate gene expression and protein translation and affect all aspects of cellular physiology. Accumulating evidence indicates that many miRNAs are aberrantly expressed in human cancers, and miRNA expression profiles have augmented prognostic information provided by traditional classification schemes related to stage and subtype [3], [4], [5]. Viruses also encode miRNAs and thereby affect functioning of infected cells. In mammals, viral infection is a potent trigger of the interferon response which inhibits viral replication and mitigates viral damage. Infection of mammalian cells by RNA viruses, except retroviruses, leads to the generation of long dsRNAs during the virus life cycle. DNA viruses produce dsRNAs by convergent transcription of their compact viral genomes. Viral dsRNA is a potent trigger of the interferon response which phosphorylates the translation factor eIF2a and leads to global translational arrest and apoptosis [6], [7], [8]. As an adaptive strategy, viruses have evolved a diverse array of countermeasures to block interferon production, and some of these rely on viral miRNAs as effectors of cellular control. All herpes viruses currently known (human and non-human) encode multiple miRNAs [9]. As an example, the hCMV miR-UL112-1 inhibits not only viral IE1 appearance but also cellular MICB expression to promote viral latency and avoid eradication by natural killer cells [10]. Therefore, it appears that herpes viruses are capable of hijacking the intracellular control of gene/protein expression through viral miRNAs.

Herpetic infections are stubbornly common and pervasive in humans. EBV [11] and CMV [12] infections are present in at least 80% of the population. Worldwide rates of Herpes simplex virus (HSV) infection, counting both cold sores (HSV-1) and genital herpes (HSV-2), are between 65% and 90% [13]. These epidemiological data imply a high probability that ovarian cancer patients are carriers of at least one or more herpetic infections. Due to their widespread prevalence and persistence and capacity to influence transcription and translation in infected cells, we hypothesize that herpes viral miRNAs are clinically important mediators of SEOC biology with significant potential as biomarkers and drug targets.

## Results

### Expression of viral miRNAs is higher in SEOC than in normal tissues

**Figure 1:**
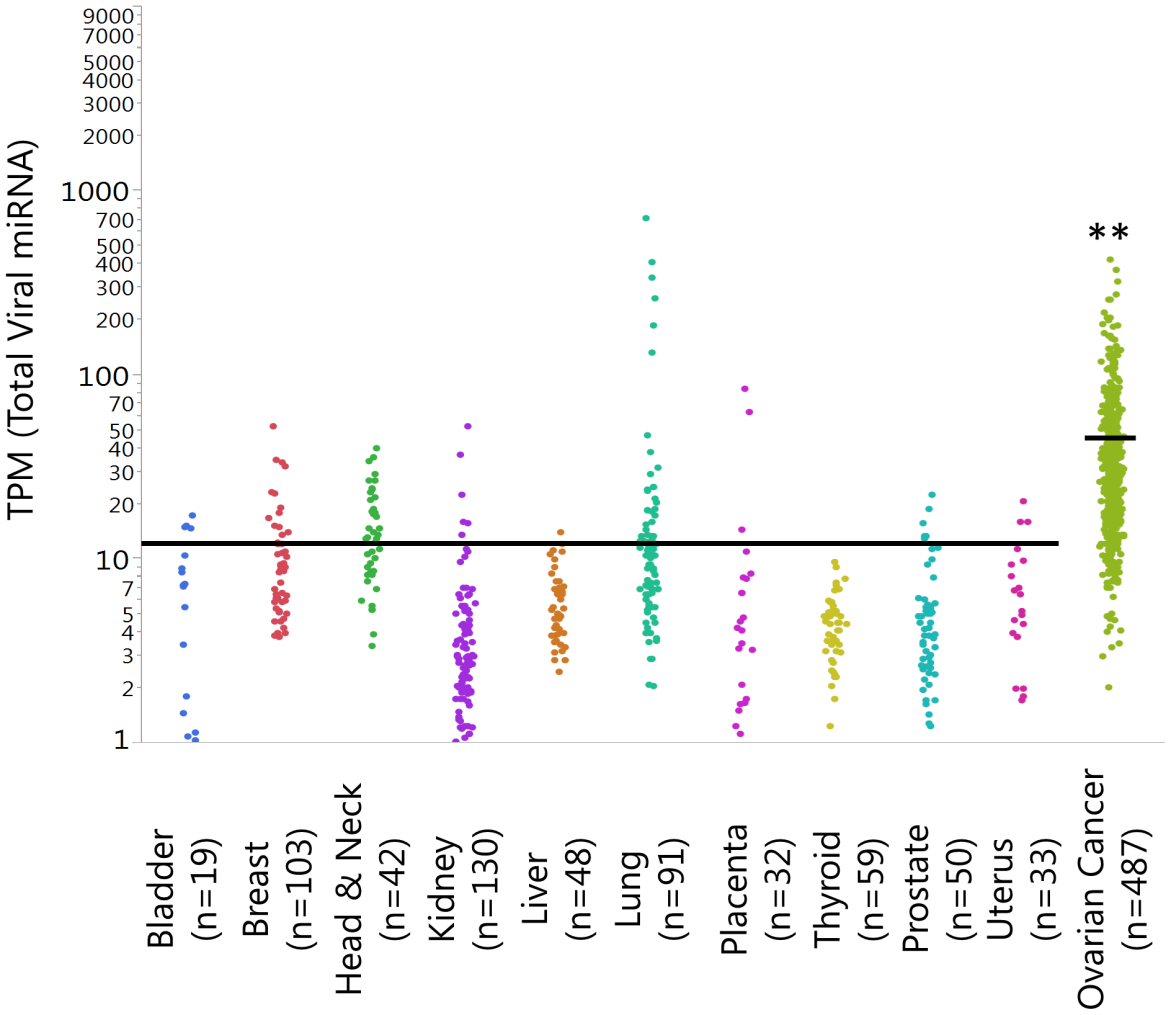
Chart showing the results of miRNA-Seq analysis in 607 noncancerous tissues and 487 SEOC patients from the TCGA dataset. Double asterisks indicate significant difference (p<0.0001, t-test) between the expression of viral miRNA in SEOC as compared to noncancerous tissues. Data are expressed to the sum of all the viral miRNAs. Data are expressed as TPM and the bar on the chart corresponds to the average of noncancerous tissues (11.8) and SEOC (45.6).

The Cancer Genome Atlas (TCGA) project [14] analyzed and catalogued messenger RNA expression, miRNA expression (Illumina Hiseq), promoter methylation and DNA copy number in 489 advanced serous ovarian adenocarcinomas and the DNA sequences of exons from coding genes in 316 of these tumors. This pioneering work is an outstanding resource for the development of new and innovative strategies for ovarian cancer treatment. The TCGA miRNA studies published to date used only the level 3 data which represents the mapped miRNAs using only the human (not viral) miRNAome as a reference. In order to overcome this limitation, we downloaded the level 1 raw data, and we performed miRNA-seq mapping for 487 patients referencing both human *and* viral miRNAomes. We were successful in mapping 88.2% of reads. The average number of mapped reads for each patient was 9.2 million. As anticipated, the major portion of reads was mapped onto the human miRNAome. However, a detectable and rather sizable number of reads (n = 184,618) was mapped onto the viral miRNAome, thus demonstrating the presence of viral miRNAs in SEOC patients. Results were normalized to take into account that the total number of reads from each patient was not identical and that, in the absence of normalization, variations in the number of reads of individual miRNA could be due to sequencing depth. Therefore we normalized data as TPM (transcripts per million) reads. The average number of viral miRNA reads for each patient was 45.6 TPM. The most abundant viral miRNAs were mapped in the HSV-1 (n = 128,701) and HSV-2 genome (n = 51,709). HH6VB and EBV accounted for 986 and 1,260 reads, respectively. CMV (Cytomegalovirus) and KSHV (Kaposi Sarcoma Herpes Virus) were present with 96 and 178 reads, respectively. TCGA does not include normal ovarian tissue controls for miRNA-seq. In order to have a reference range for the expression of viral miRNAs in noncancerous tissues, we downloaded 607 normal tissues from the TCGA including bladder, breast head &neck, kidney, liver, lung, placenta, thyroid, prostate and uterus for a total of 7.7 billion of sequences. Specimens were analyzed following the same procedure described above. In noncancerous tissues, the viral miRNA levels averaged significantly lower (TPM 11.8), as compared with a TPM mean of 45.6 in the SEOC (Fig. 1). These findings demonstrate that the expression of viral miRNAs is higher in SEOC than in noncancerous tissues.

**Figure 2:**
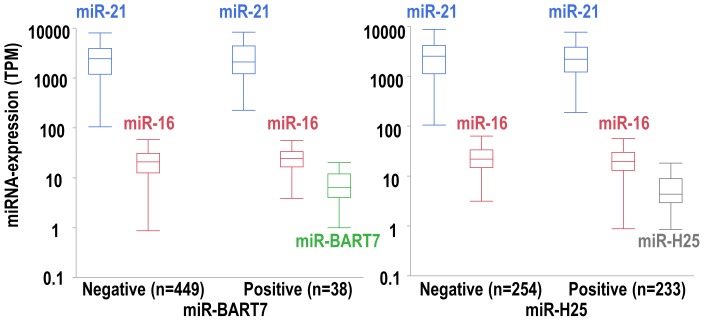
Box-whisker plot showing the expression of miR-21 (blue), miR-16 (red), miR-BART7 (green) and miR-H25 (gray) in 487 SEOC patients of the TCGA study. Women were categorized as negative or positive for miR-BART7 and miR-H25 if TPM >0 or TPM  = 0, respectively. In each plot the horizontal line corresponds to the median, the box to the 25^th^-75^th^ percentile and the lines to the confidence interval (5^th^–95^th^ percentile).

Thereafter, we performed a comparative analysis of the expression levels of both miR-H25 and miR-BART7 and some human miRNAs typically expressed in the epithelial component of SEOC (miR-21) [15], [16] and in red blood cells (miR-16) [17] (Fig. 2). MiR-21 expression levels were significantly higher than those of miR-16 (p<0.001, paired t-test). A similar pattern was also observed for both miR-H25 and miR-BART7, which were expressed at significant lower levels in comparison to miR-21 (p<0.001, paired t-test). As compared with miR-16, again both viral miRNAs were significantly expressed at lower levels (p<0.001, paired t-test), even if in this case the difference in the expression was less consistent than that noticed for miR-21.

### Prognostic role of viral miRNAs in SEOC

**Figure 3:**
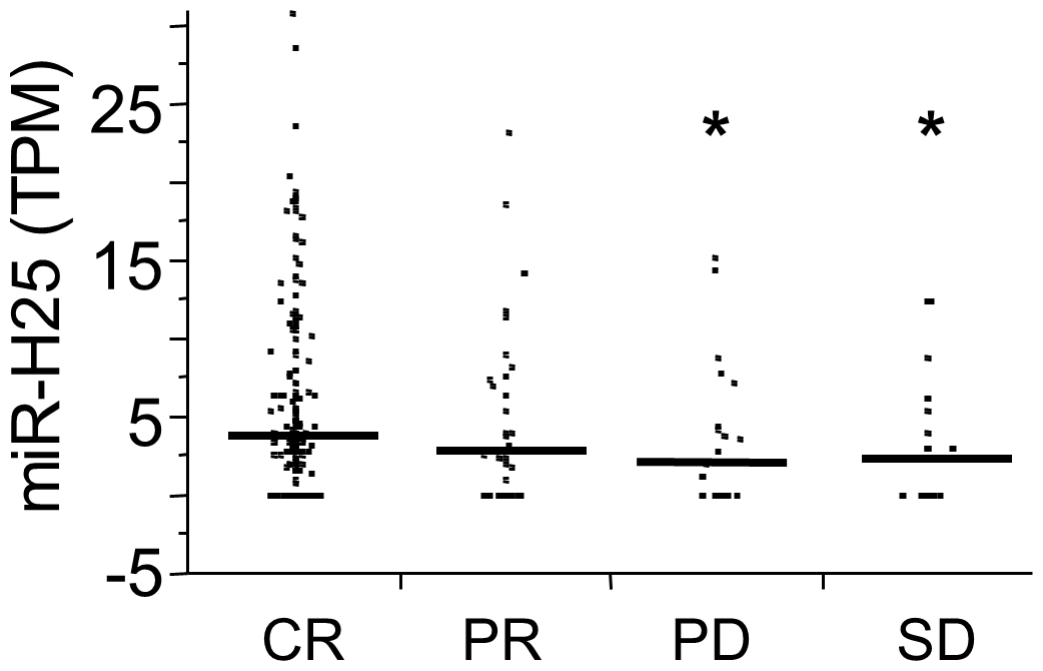
Expression of miR-H25 in 487 ovarian cancer patients stratified according to clinical response defined as CR (complete response), PR (partial response), PD (progressive disease) and SD (stable disease). Asterisks mark significant decreases of the expression of miR-H25 in patients with both PD and SD as compared with patients with CR. This finding supports a protective role of miR-H25 expression.

In order to assess whether the expression of viral miRNAs was prognostic, we analyzed each individual viral miRNA in a Cox regression model. Analysis was performed in univariate and multivariate analysis including age and stage, since these variables were significant univariate predictors. The endpoint was overall survival (OS) measured in months. A hazard ratio (HR) >1 indicated a detrimental effect on OS, while HR<1 signified a protective effect. Analysis was performed using the expression of viral miRNA (TPM) as a continuous variable. The total pooled analysis for each virus did not provide significant predictive capability in the Cox multivariate model (*p*>0.05). Only HSV-1 trended toward a significant protective effect with a *p*-value in multivariate analysis of 0.053 (HR = 0.33; CI 0.03–1.01). When limiting the pool, however, to include only those HSV-2 viral miRNAs typically expressed during productive infection (miR-H9, miR-H11, miR-H12, miR-H13, miR-H19, miR-H20, miR-H21, miR-H22, miR-H23 and miR-H25 [9]), the protective effect reached statistical significance (HR = 0.2, CI 0.04–0.89, *p* = 0.032). Notably, the expression of viral miR-H25 (HSV-2) was found in 233/487 (48% with TPM >0) of the specimens. Multivariate analysis utilizing the Cox model showed that high expression of this viral miRNA was related to a better outcome (HR = 0.1; CI 0.01–0.55; *p* = 0.006). Additionally, the expression of miR-H25 was higher in patients with complete chemotherapy response and lower in patients with progressive disease (Fig. 3). Although the mechanism is unknown, productive infection of HSV-2 with associated high expression of miR-H25 may inhibit cancer cell replication directly or increase killing of HSV-2 infected cancer cells by the immune system.

**Figure 4:**
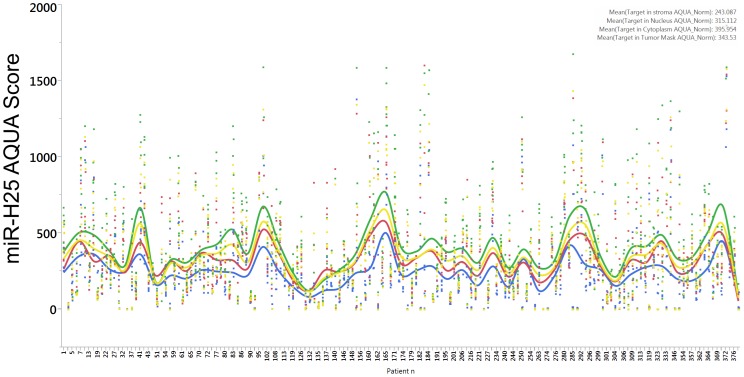
Dot chart reporting the AQUA scores of miR-H25 expression in 161 SEOC patients. Each dot corresponds to the value of an individual tissue core. AQUA scores were calculated in the stromal mask (blue dots), in the nuclear mask (red dots), in the tumor cytoplasmic mask (green dots) and in the tumor mask (yellow dots). The corresponding lines refer to the smoothed average for each patient and the coloring is the same of the dots.

**Figure 5:**
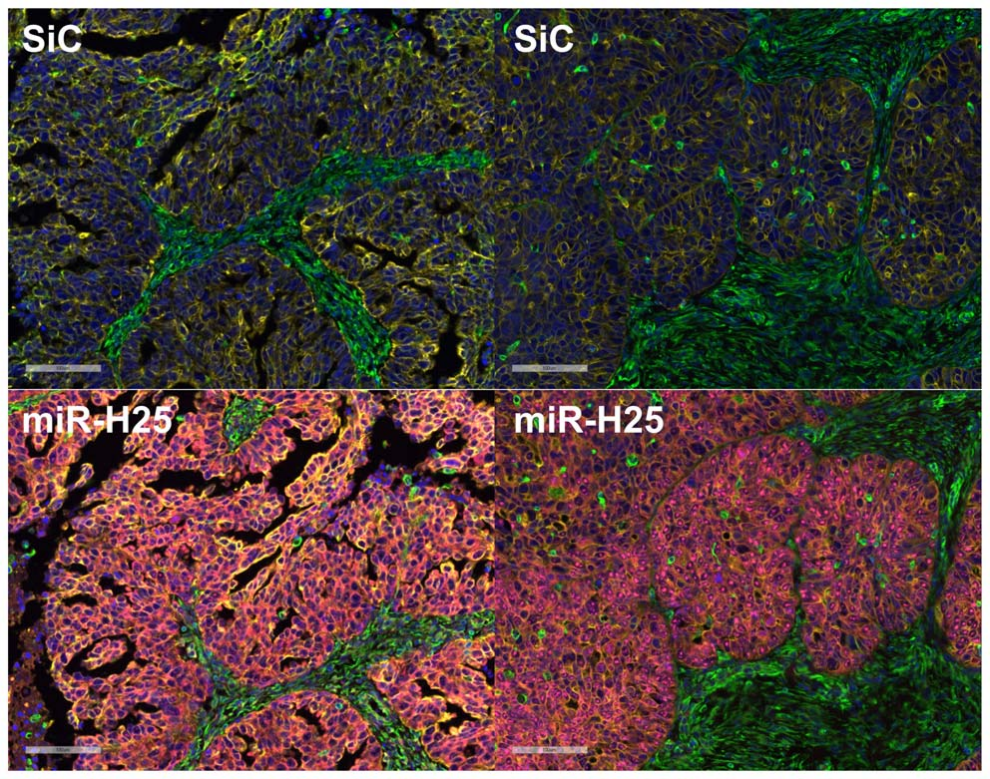
Representative merged images depicting four channel fluorescent immunohistochemistry and in situ hybridization in two consecutive sections of the same patient. At top SiC probe and at bottom miR-H25 probe. Blue signal  =  DAPI. Yellow  =  Cytokeratin. Green  =  Vimentin. Pink  =  miR-H25 probe. Bar size equals 100 µM.

**Figure 6:**
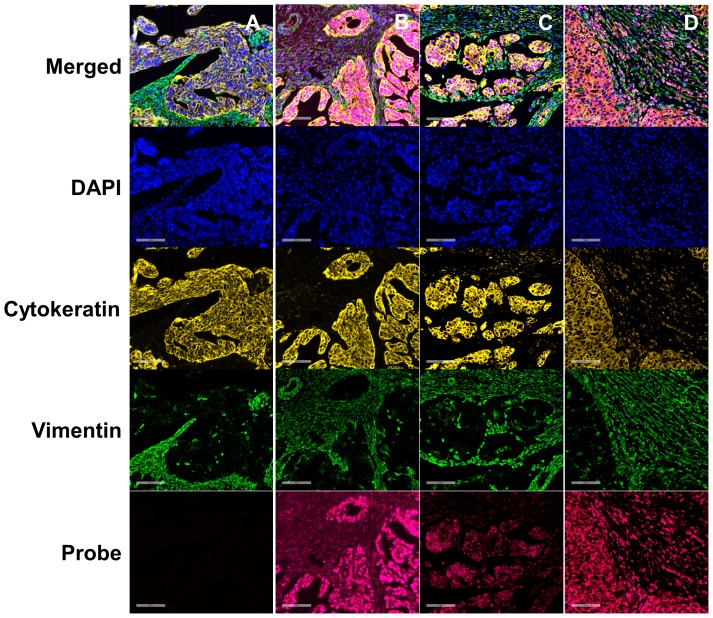
Representative images depicting four channel fluorescent immunohistochemistry and in situ hybridization. From top to bottom: merged image, nuclear staining (DAPI), tumor mask (cytokeratin immunostain), stromal mask (vimentin immunostain) and RNA probe. Probes are SiC (scrambled interfering control) (A), U6 (positive control) (B), and miR-H25 (C & D). C represents the expression of miR-H25 typical of latent HSV-2 infection (nuclear staining of tumor cells). D represents expression of miR-H25 characteristic of productive HSV-2 infection (cytoplasmic expression in tumor and stroma). Bar size equals 100 µM.

Thereafter, we analyzed the expression of miR-H25 in an additional cohort of 161 SEOC patients whose clinical features are summarized in table 1. For each patient, up to 12 tissue cores were taken from different regions of each cancer (933 cores in total), and these were interrogated in a tissue micro array (TMA) format. The expression of miR-H25 (stained with custom made Exiqon probe) in each core was analyzed with AQUA software which utilizes a predefined set of algorithms and unsupervised method to assess target levels in various cellular sub-compartments (or “masks”). Cytokeratin and vimentin antibodies staining provided the tumor and stromal masks respectively, and DAPI served to define the nuclear mask. A summary of the results of AQUA scoring is reported in Fig. 4. Overall intensity of miR-H25 was higher in the cytoplasm of cancer cells and lower in the stromal mask. Negative and positive controls were obtained using an oligonucleotide not targeting any region of the human genome (SiC) and the U6 small RNA, respectively. SiC probe showed no staining (Figs. 5 & 6A). As expected, the U6 probe demonstrated nuclear expression in almost 100% of the cells (Fig. 6B). Expression of U6 was higher in the epithelial cancer cells as compared with the levels noticed in stromal cells (Fig. 6B). MiR-H25 probe was detected with two patterns of expression. In one, the probe staining was confined to the nucleus of tumor cells (Fig. 6C). In a second, there was diffuse cytoplasmic staining of both tumor and stromal cells (Fig. 6D). Additional figures to describe at higher magnification the staining pattern are provided as S1, S2, S3, S4 and S5 Figures. Multivariate Cox analysis including age and stage revealed that the expression of nuclear miR-H25 was not predictive of outcome (HR 0.91, CI 0.65–1.24), whereas the expression with a cytoplasmic staining pattern was significantly associated with good outcome (HR 0.56; CI 0.39–0.79; *p* = 0.0008). This AQUA analysis fully supports our discovery from TCGA dataset, namely that productive HSV-2 infection (with concomitant cytoplasmic miR-H25 over-expression) offers protection to SEOC patients.

**Table 1:** Clinical Features of the analyzed setting of ovarian cancer patients.

Characteristics	Number (%)
**Cases**	161
**Age, yrs Median (range)**	60 (25–84)
**FIGO Stage**	
**I–II**	10 (6.1)
**III**	131 (81.5)
**IV**	20 (12.4)
**Histotype**	
**Papillary-serous**	161 (100)
**Ca 125 Median (range)**	617 U/mL (11–34000)
**Status**	
**Dead**	116 (72.0)
**Alive**	45 (28.0)
**Median follow up (alive)**	54 months

**Figure 7:**
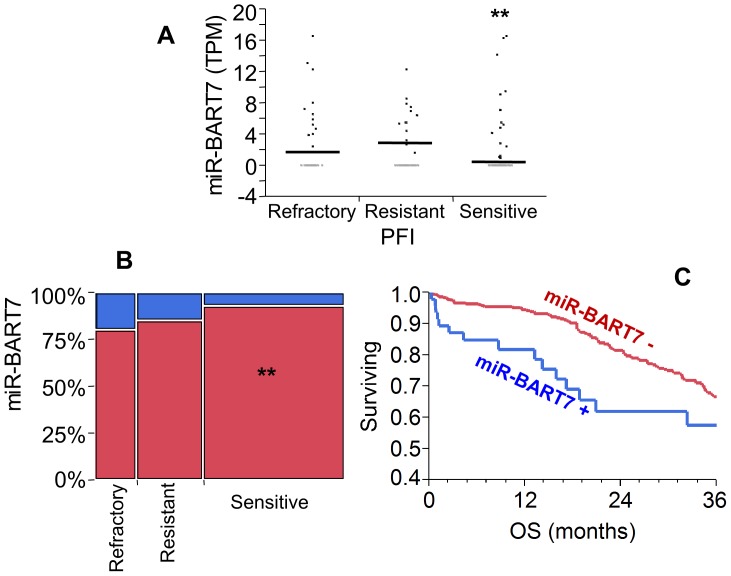
Analysis of miR-BART7 expression according to response to chemotherapy. A: Patients were labeled according to PFI as refractory (PFI <6 months), resistant (PFI 6-12 months) and sensitive (PFI >12 months). Expression of miR-BART7 is significantly lower in the sensitive as compared to refractory and resistant groups (double asterisk, p<0.001, t-test). B: Contingency analysis (mosaic plot) of patients grouped for expression of miR-BART7 (TPM >0 is positive (n = 38), blue bars; TPM  = 0 (n = 449) is negative, red bars) and according to response to chemotherapy as described in A. Double asterisks show that the proportion of sensitive patients is higher in the miR-BART7 negative group (Fisher exact test, p<0.001, double asterisks). C: Kaplan-Meier analysis of the TCGA patients (n = 487) according to the expression of miR-BART7. The early mortality rate is significantly higher in miR-BART7 positive patients (Wilcoxon test, p = 0.01). OS (X axis) represents overall survival expressed in months.

By contrast, TCGA miRNA-sequencing analysis demonstrated that expression of miR-BART7 (produced by EBV) was related to shortened PFI (platinum free interval) and poor outcome. Although expression of miR-BART7 (TPM >0) was identified only in 7.9% of the samples overall, it was over-represented in patients with refractory and resistant disease as compared with the chemo-sensitive group (Fig. 7A and 7B). Accordingly, miR-BART7 positive patients exhibited shortened overall survival in Kaplan Meier (Fig. 7C) and Cox multivariate analysis (HR 3.4, CI 2.2–6.6, *p* = 0.002).

### Identification of modifier mechanisms of SEOC biology

**Figure 8:**
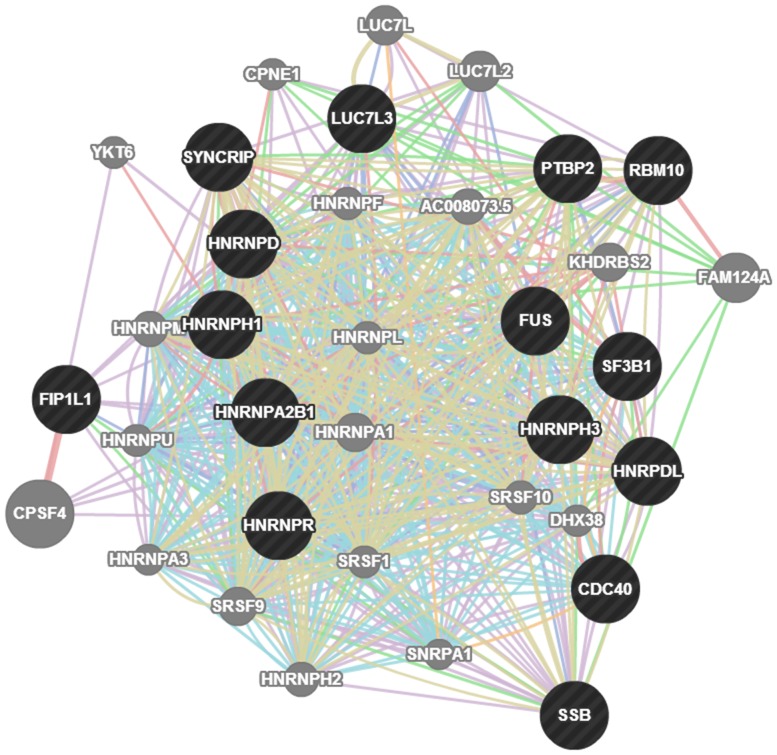
Interaction map of the genes predicted as targets of miR-H25. The miR-H25 network shows 35 genes involved in the RNA processing, splicing and transport. Coverage of the network in the DAVID database is 15/35 (42%, black circles).

**Figure 9:**
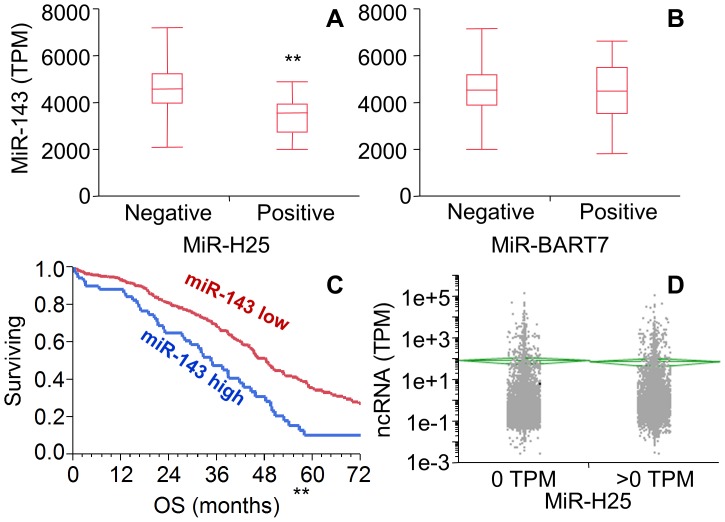
Box-whisker plot chart showing the expression of miR-143 (TPM) according to expression of miR-H25 (A) and miR-BART7 (B). Patients were defined as positive for viral miRNA expression if the TPM value was >0 (n = 233). Patients with TPM  = 0 were defined negative (n = 254). The chart shows a significant downregulation of miR-143 in women positive for miR-H25 (p<0.001, t-test) but not in patients positive for miR-BART7. Kaplan-Meier analysis of patients according to the expression of miR-143 (C). Patients with high expression of miR-143 exhibit a significantly worse outcome compared to patients with low miR-143 expression (P<0.001, Wilcoxon test). D: Analysis of expression of non-coding RNA (as TPM) in patients grouped for miR-H25 expression. Green lines and diamonds depict the means and the confidence intervals of the means of the two groups. Difference is not statistically significant (*p*>0.05, t-test).

**Figure 10:**
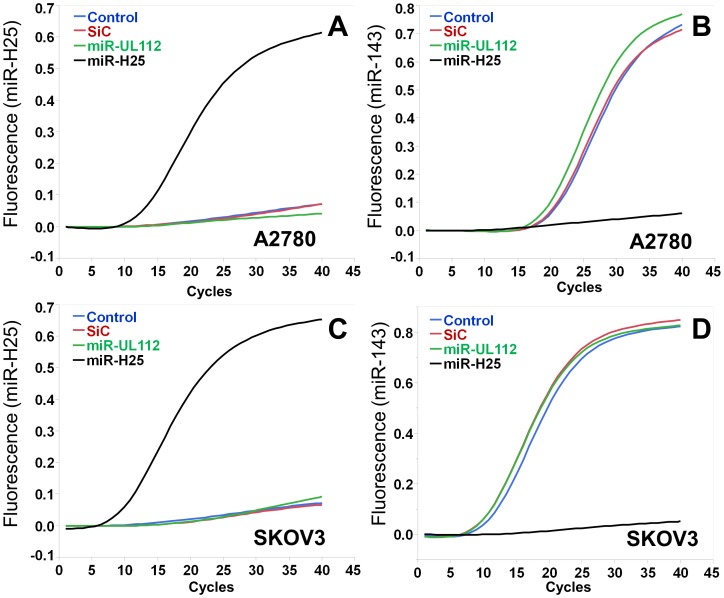
Representative qPCR analysis in A2780 and SKOV3 cells with transfection of a synthetic miR-H25. In A–C and B–D the expression of miR-H25 and miR-143, respectively. In A–B and C–D, A2780 and SKOV-3 cells, respectively. Blue: control with transfecting medium; Red: Scrambled control synthetic probe (SiC); Green: miR-UL112 synthetic; Black: miR-H25 synthetic. Data show that the expression of miR-H25 (10 nM) suppresses miR-143 expression in both cell lines.

One of the advantages of the TCGA dataset is its inclusion of both miRNA-seq and gene expression data. This feature enables performance of integrated analyses aimed at identifying candidate genes regulated by viral miRNAs as previously described for human miRNAs [18]. We had focused our analyses on the two individual viral miRNAs (miR-H25 and miR-BART7) which showed significance in clinical outcome studies as described above. We downloaded the level 2 data reporting gene expression analyses utilizing Affymetrix U133 chips. For 414 patients (who were randomly assigned to either a training or testing set), we successfully analyzed both gene and viral miRNA expression. We grouped patients according to the expression levels of the two viral miRNAs of interest (positive  =  TPM >0 vs. negative  =  TPM  = 0). The genes significantly different between these two groups were identified at a confidence level of *p*<0.05 after multiple hypothesis correction with the Benjamini-Hochberg method. Using this approach, we found 262 genes differentially expressed for miR-H25. According to the DAVID bioinformatic resource [19], they clustered into 12 independent functional groups, with RNA-processing, splicing and transport as the most significant (Fig. 8). The impact on RNA processing was accompanied by a significant modulation of the expression of some human miRNAs. For example, women with high levels of miR-H25 (Fig. 9A) but not miR-BART7 (Fig. 9B) showed a significant reduction in the expression of miR-143. MiR-143 is a potent miRNA, which, in the TCGA study, was related to poor outcome in the multivariate Cox model (HR 14.8, CI 5.9–37.9, *p*<0.001) and Kaplan-Maier analysis (Fig. 9C). The effect of miR-H25 on miR-143 seems unrelated to nonspecific down-regulation of miRNA processing, as no significant difference between miR-H25 negative and positive patients in the overall expression of non-coding RNA was observed (Fig. 9D). The influence of miR-H25 on miR-143 expression may, in fact, be specific and direct; we were able to reproduce the same phenomenon in vitro utilizing two SEOC cell lines (A2780 and SKOV-3) transfected with a synthetic miR-H25. Over-expression of miR-H25 was tested at three concentrations (0.1, 1 and 10 nM) using the transfecting medium, a scrambled oligo not targeting any region of the human genome (SiC) and a viral miRNA not expressed in SEOC (miR-UL112) as negative controls. Only miR-H25 produced a significant down-regulation of miR-143 expression (Fig. 10).

**Figure 11:**
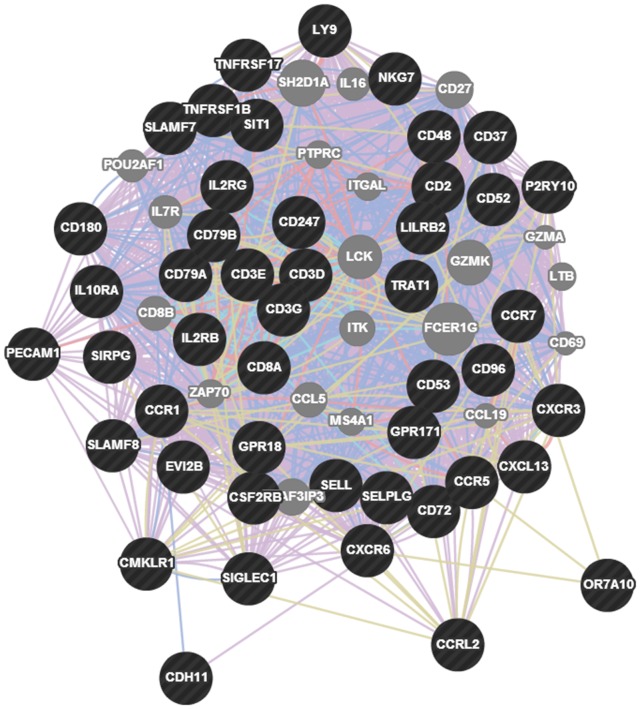
Interaction map of the genes predicted as targets of miR-BART7. The miR-BART7 network shows 67 genes involved in T cell activation. Coverage of the network in the DAVID database is 47/67 (70%, black circles).

**Figure 12:**
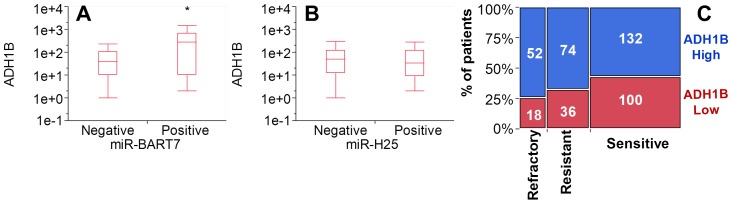
Box-whisker plot chart showing the expression of ADH1B according to expression of miR-BART7 (A) and miR-H25 (B). Asterisk indicates a significant overexpression of ADH1B in miR-BART7 positive patients (*p* = 0.02, t-test) but not in miR-H25 positive patients. Contingency analysis (C) (mosaic plot) of patients stratified according to ADH1B expression (blue and red are for high and low expression, respectively) and sensitivity to chemotherapy (refractory, resistant and sensitive categories as described in Fig. 5A). ADH1B expression is significantly higher in refractory and resistant patients (*p*<0.05, Fisher exact test) as compared with the sensitive group (similar to miR-BART7).

**Figure 13:**
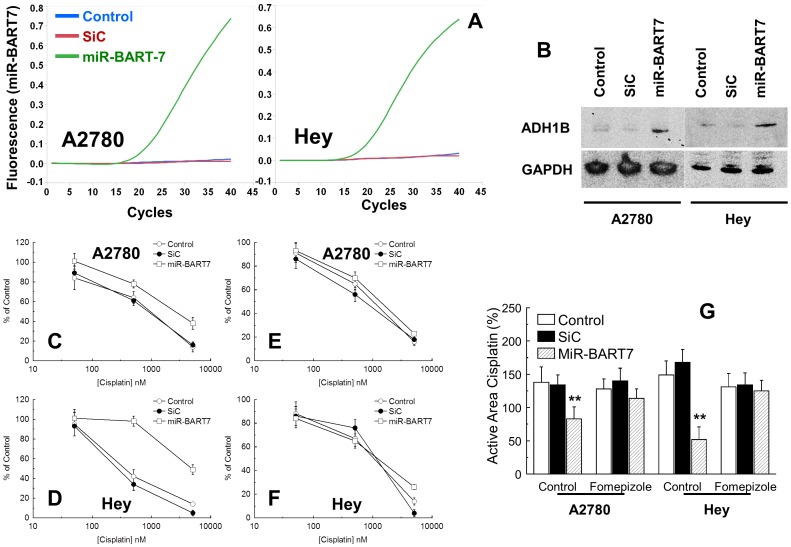
Representative qPCR analysis of A2780 cells transfected with a synthetic miR (10 nM) corresponding to the sequence of miR-BART7 (green line) (A). Controls were represented by cells treated with only the transfecting medium (blue line) and a sequence not targeting the human genome (SiC, red line). Representative western blot in cells treated as in (A). Treatment with the synthetic miR-BART7 induced the expression of ADH1B at the protein level in both cell lines. GAPDH served as loading control (B). Line chart showing the effect of cisplatin in A2780 (C) and Hey (D) cells transfected as described in (A). Line chart showing the effect of cisplatin plus fomepizole (10 µM) in A2780 (E) and Hey (F) cells transfected as described in (A). Bar chart (G) showing the active area of cisplatin in A2780 and Hey cells. Cells were treated as in (D–E). Cisplatin effects were monitored after 72 hours of culture in the presence (or not) of fomepizole 10 µM. Bars and error bars correspond to mean and SD of triplicate samples performed in duplicate. Double asterisks mark a significant effect at a *p*<0.001 level (t test).

For that concerning miR-BART7, we performed the same gene network analysis described above for miR-H25. MiR-BART7 expression clustered with 221 genes in 6 functional groups, the most prominent of which is the T cell activation pathway (Fig. 11). Also, we identified a significant upregulation of the ADH1B gene. This gene encodes for Alcohol Dehydrogenase 1B, the key enzyme for the conversion of ethanol into acetaldehyde, a substance which interferes with DNA repair [20]. ADH1B was significantly increased in women expressing miR-BART7 (Fig. 12A) while it was unchanged in women expressing miR-H25 (Fig. 12B). Supporting the miR-BART7 prognostic data presented in Fig. 5, women with high expression of ADH1B were relatively over-represented among patients refractory or resistant to first-line chemotherapy (Fig. 12C). In order to explore the connection between miR-BART7 expression and resistance to chemotherapy, we transfected A2780 and Hey cells (two cell lines cisplatin-sensitive) with a synthetic miR-BART7 (10 nM, Fig. 13) or a scrambled control (SiC). Efficiency of transfection was confirmed with qPCR (Fig. 13A). The effects of miR-BART7 were also assessed in terms of ADH1B induction at the protein level with the western blot technique (Fig. 13B). MiR-BART7 produced a significant increased expression of ADH1B in both cellular models. Increase was quantified with densitometric analysis in three independent experiments and was 4.5 and 3.2 in A2780 and Hey cells, respectively (*p*<0.001 t-test). After 12 hours from transfection, cells were incubated with cisplatin for 72 hours and then counted. In order to quantify the results we used the cisplatin active area (%) as recently described by Barretina and coll. [21]. Transfection with miR-BART7 produced a significant increase in cisplatin-resistance in both cell lines (Fig. 13C and Fig. 13D). As noticed in patients at the gene level, miR-BART7 induced a significant increase of ADH1B expression in both cell lines. Moreover, we tested the effect of fomepizole, a known specific inhibitor of ADH1B activity [22]. Fomepizole completely abrogated the effect of miR-BART7 on cisplatin toxicity (Fig. 13E, 13F and 13G), thus demonstrating that miR-BART7 diminishes cisplatin efficacy by increasing ADH1B activity.

## Discussion

SEOC is the deadliest gynecologic malignancy. Patients will benefit from the identification of biomarkers useful for diagnosis of early (more treatable) disease, and from the development of targeted therapy. This study is, to our knowledge, the first detailed investigation of the role of viral miRNA expression in ovarian cancer. First, we demonstrate that the expression of total viral miRNAs is higher in SEOC as compared with normal tissues. As a part of the strategies adopted by ovarian cancer cells to escape the recognition by the immune system, there is the inhibition of endogenous interferon response [23], [24], [25]. The insufficient interferon response creates a cellular environment supportive for viral growth and replication [8], thereby explaining the increased levels of viral miRNAs in SEOC tissues. Since miRNAs are resistant to degradation and can be detected in peripheral blood [26], we hypothesize that circulating total viral miRNAs will be higher in the ovarian cancer population compared to controls. A limitation of our investigation is that in the TCGA dataset there are no normal controls coming from ovarian tissues. However, we took in consideration the normal expression levels obtained in a large panel of tissues. Across all these noncancerous tissues there was no significant difference in the expression of viral miRNAs. Therefore, we believe extremely unlikely that in ovarian normal tissue for some reasons there is an increased expression of viral miRNA.

Second, we report that two individual viral miRNAs miR-H25 and miR-BART7, act as modulators of SEOC biology. HSV-2 productive infection induces a high level of miR-H25 [27] that correlates with a better outcome. We document this apparent protective effect in two independent clinical cohorts utilizing two independent approaches, miRNA-sequencing (TCGA) and quantitative immunofluorescence (AQUA). AQUA allows cellular subcompartment analysis, and this method reveals that the protective effect of miR-H25 is evident only when its expression is cytoplasmic (and characteristic of productive HSV-2 infection) rather than nuclear (implying latent HSV-2 infection). MiR-H25 may exert its effects by hijacking host RNA processing and down-regulating human miR-143, a potent miRNA in human cellular function [28]. Indeed, we provide direct evidence for this phenomenon in cells transfected with a synthetic miR-H25. Although, the use of oncolytic HSV strains as a treatment for ovarian cancer patients has been previously reported [29], [30], [31], the strategy entailed vaccination with the aim of limiting productive HSV infection. Our observations suggest, by contrast, that productive HSV-2 infection is potentially advantageous and could be exploited to increase the efficacy of oncolytic HSV strains.

Whereas miR-H25 provides an apparent protective effect, miR-BART7 is associated with high early mortality. In keeping with recent findings in nasopharyngeal carcinoma [32], we show a role for miR-BART7 in inducing shortened platinum free interval (PFI) in a small subset of patients in whom this miRNA is expressed. Transfection studies with a synthetic miR-BART7 produced a significant increase in cisplatin-resistant cells which is fully reverted with the use of fomepizole, a known inhibitor of ADH1B. In our analysis, both miR-BART7 and ADH1B are elevated in patients refractory or resistant to first line chemotherapy. The relationship between miR-BART7 and ADH1B is of interest due to the report of ADH1B as a potential source of chemoresistance in ovarian cancer [33]. Fomepizole is currently approved for the treatment of methanol and ethylene glycol intoxications with an apparent excellent toxicological profile [22]. Thus, it looks plausible that, in patients with high levels of miR-BART7, ADH1B inhibition may result in a better response to cisplatin.

Our study is the first report showing a potential involvement of viral miRNA as drivers of ovarian cancer biology. We can explain our findings with three nonexclusive pathogenetic models. In the first, viral infection occurs in the ovarian cancer cells with concomitant production of viral miRNA. We believe this possibility unlikely, for the high difference in the expression levels of the viral miRNAs as compared with human miRNA expressed in cancer cells like miR-21. In the second, the infection occurs in the ovarian tissue in stromal cells where it is known that the virus can replicate such as B and neural cells for EBV and HSV-2, respectively. A third model is that the infection is not occurring in the ovarian tissue but in a remote site and the viral miRNA are taken by the cancer through circulation.

In conclusion, we provide compelling evidence for the role of herpesviruses encoded miRNAs in SEOC. Our observations that total viral miRNA and at least two specific viral miRNAs are increased in ovarian cancer patients may aid in the development of biomarkers of early disease and formulation of novel anticancer strategies.

## Material and Methods

### Bioinformatic analysis of TCGA data

Level 1 high-throughput miRNA sequencing data from 487 serous ovarian cancer patients profiled using Illumina HiSeq sequencer were downloaded from the TCGA data portal to local servers. High-throughput sequencing data were analyzed according to the pipeline created on CLC genomics workbench 6.5.1 software (CLC GW). Level 2 RNA expression data (HT-HG_U133A) were retrieved from the TCGA data portal. Alignment between miRNA-seq and RNA expression data was possible for 412 patients. Specimen barcodes of the noncancerous setting are reported in S1 Table. Detailed pipeline description is provided in S1 Document.

### Statistical Analysis

The significance of increased/decreased expression of viral miRNAs or other variables has been computed with the use of t-test or Fisher exact test of numeric or categorical variables. For categorization of numerical variables in the case of viral miRNAs, expression negative and positive were 0 and >0 TPM, respectively. In the case of miR-143 and ADH1B, a cutoff value has been optimized after setting a ROC curve as previously described [34]. Outcome analysis was performed using the expression of viral miRNAs as numeric and overall survival in months in a multivariate Cox model including age and stage. For Kaplan-Meier analysis, Wilcoxon test was used to test if the difference in survival was statistically significant across two categorical groups. Type I error for all the tests was set at 5%, and all the analyses have been performed with JMP9.

### Patients and Clinical Samples

Primary tumor tissues from anonymized patients diagnosed with serous ovarian carcinoma at the Catholic University of Rome (Italy) were used in this retrospective study. All the patients provided written consent and the protocol study was approved by the Danbury Hospital IRB (DH17/12). In total, 161 patients were registered from the hospital database. Features of the clinical setting are summarized in Table 1.

### Tissue Microarray (TMA) Construction

All SEOC cases were reviewed by 2 pathologists (S.S. and P.F.), and the most representative areas of tumor cells were carefully selected and annotated on sample slides. Corresponding paraffin blocks were sampled with three replicate 2-mm cores of tumor tissue. In addition, several normal tissues were included in the TMA construction to serve as controls. Multiple 6-µm sections of TMA blocks were cut and used for in situ hybridization and immunohistochemical analysis.

### Quantitative In Situ Hybridization (ISH)

Double-DIG labeled (Exiqon, Copenhagen, Denmark) miRCURY LNA detection probes were used for visualization of the miRNA hsv2-miR-H25 and included a scrambled probe as negative control and U6 as a positive control. Briefly, slides were hybridized for 1h at 55°C with 10nM Double Digoxigenin (DIG) LNA modified probe for Mir-H25 (Sequence: 5′-CGGCGGCGACCGGGA-3′), 40 nM for the scrambled probe SiC (Sequence: 5′-GTGTAACACGTCTATACGCCCA-3′) and 1 nM for U6 Probe (Sequence: 5′-CACGAATTTGCGTGTCATCCTT-3′). Slides were then stringently washed once in 5X SSC (Saline Sodium Citrate: 3 M NaCl, 0.3 M Sodium citrate) buffer, twice in 1X SSC, and twice in 0.2X SSC at hybridization temperature, and then once at room temperature in 0.2X SSC. The slides were incubated with blocking solution for 15 min at RT and then with Anti-Digoxigenin-POD, Fab fragments from sheep (Roche Diagnostics) diluted 1∶100, rabbit anti-cytokeratin (Dako Corp, Carpinteria, CA, USA) diluted 1∶100 and chicken anti-vimentin (Millipore Corp, Billerica, MA, USA) diluted 1∶200 in the antibody dilutant solution for 1 h at room temperature. Following two washes with 0.1% Tween PBS (PBS-T) and one wash in PBS for 5 min each, the miRNA signal was detected with the TSA Plus Cyanine 5 system (Perkin Elmer, Norwalk, CT, USA), the slides were washed again with PBS-T and PBS as above, and cytokeratin was detected with Alexa 555-conjugated goat anti-rabbit secondary antibody and vimentin with Alexa 488- conjugated goat anti-chicken secondary antibody (Life Technologies Corp, Carlsbad, CA) diluted 1∶100 in PBS for 30 min. The slides were mounted with Prolong mounting medium containing 4′,6-Diamidino-2- phenylindole (DAPI, Molecular Probes, Eugene, OR, USA).

Images were automatically acquired with Aperio Scanscope FL and then analyzed using the AQUA software (Genoptix).

### Expression of synthetic miR-25 and miR-BART7

Biotinylated miR-H25 and miR-BART7 were obtained from Eurofins MWG Operon as a miRNA duplex in which the sense filament, at the 3′ end, was labeled with biotin. A detailed map of all the synthetic miR is provided in S6 Figure. A2780 and SKOV3 cells were used with miR-H25, while A2780 and Hey cells were utilized with miR-BART7. Cells were seeded in 6 well dishes, 2×10^6^ cell/well, for 48 h without reaching full confluency. HiPerFect transfection reagent (Qiagen, Valencia, CA) was used to transfect the cells at final concentration of 5–10 nM. For each cell line, a transfection with only HiPerFect reagent was performed as negative control. Analysis was carried out using the 48.48 dynamic array (Fluidigm Corporation, CA, USA). Cytotoxicity assays were performed with the use of the ATPlite kit as previously described [35]. Q-PCR analysis was performed as previously described [36]. Western blot for ADH1B expression was performed as previously described [37] using a rabbit polyclonal antibody (Thermo Fisher Scientific Pierce, Rockford, IL). A mouse anti-GAPDH (clone G-9, Santacruz Biotechnology, Dallas, TX) antibody was used as loading control.

## Supporting Information

S1 Figure Representative staining of miR-H25 in SEOC patients. In A composited image reporting the nuclei staining (blue), the tumor mask in yellow (cytokeratin), the stromal mask in green (vimentin) and the miR-H25 signal in pink. The region identified in white corresponds to the stromal tissue. In B the same image reporting only the nuclei staining (blue) and the miR-H25 signal (pink). Inside the white region (stromal tissue) the pattern of miR-H25 staining is cytoplasmic while in the epithelial cancer the staining is nuclear.
(TIF)

S2 Figure Representative staining of miR-H25 in SEOC patients. In A composited image reporting the nuclei staining (blue), the tumor mask in yellow (cytokeratin), the stromal mask in green (vimentin) and the miR-H25 signal in pink. The region identified in white corresponds to the stromal tissue. In B the same image reporting only the nuclei staining (blue) and the miR-H25 signal (pink). Inside the white region (stromal tissue) the pattern of miR-H25 staining is cytoplasmic while in the epithelial cancer the staining is nuclear.
(TIF)

S3 Figure Representative staining of miR-H25 in SEOC patients. In A composited image reporting the nuclei staining (blue), the tumor mask in yellow (cytokeratin), the stromal mask in green (vimentin) and the miR-H25 signal in pink. In B the same image reporting only the nuclei staining (blue) and the miR-H25 signal (pink). In cancer cells it is evident a bright cytoplasmic pattern of staining.
(TIF)

S4 Figure Representative staining of miR-H25 in SEOC patients. In A composited image reporting the nuclei staining (blue), the tumor mask in yellow (cytokeratin), the stromal mask in green (vimentin) and the miR-H25 signal in pink. The region identified in white corresponds to the stromal tissue. In B the same image reporting only the nuclei staining (blue) and the miR-H25 signal (pink). Inside the white region (stromal tissue) the pattern of miR-H25 staining is barely detectable, while in the epithelial cancer the staining is bright with a cytoplasmic pattern.
(TIF)

S5 Figure Representative staining of miR-H25 in SEOC patients. In A composited image reporting the nuclei staining (blue), the tumor mask in yellow (cytokeratin), the stromal mask in green (vimentin) and the miR-H25 signal in pink. The region identified in white corresponds to the stromal tissue. In B the same image reporting only the nuclei staining (blue) and the miR-H25 signal (pink). Inside the white region (stromal tissue) the pattern of miR-H25 staining is cytoplasmic while in the epithelial counterpart no miR-H25 is noticeable.
(TIF)

S6 Figure Schematic design of synthetic miR-H25 and miR-BART7.
(TIF)

S1 Table List of the TCGA barcodes for all the utilized noncancerous tissue control.
(XLSX)

S1 Document Detailed description of the sequencing pipeline.
(DOCX)
