# Supplementary figures and images for: Herpes Virus MicroRNA Expression and Significance in Serous Ovarian Cancer
Source: PLoS One. 2014 Dec 8;9(12):e114750. doi: 10.1371/journal.pone.0114750 (PMC4259392; doi:10.1371/journal.pone.0114750)

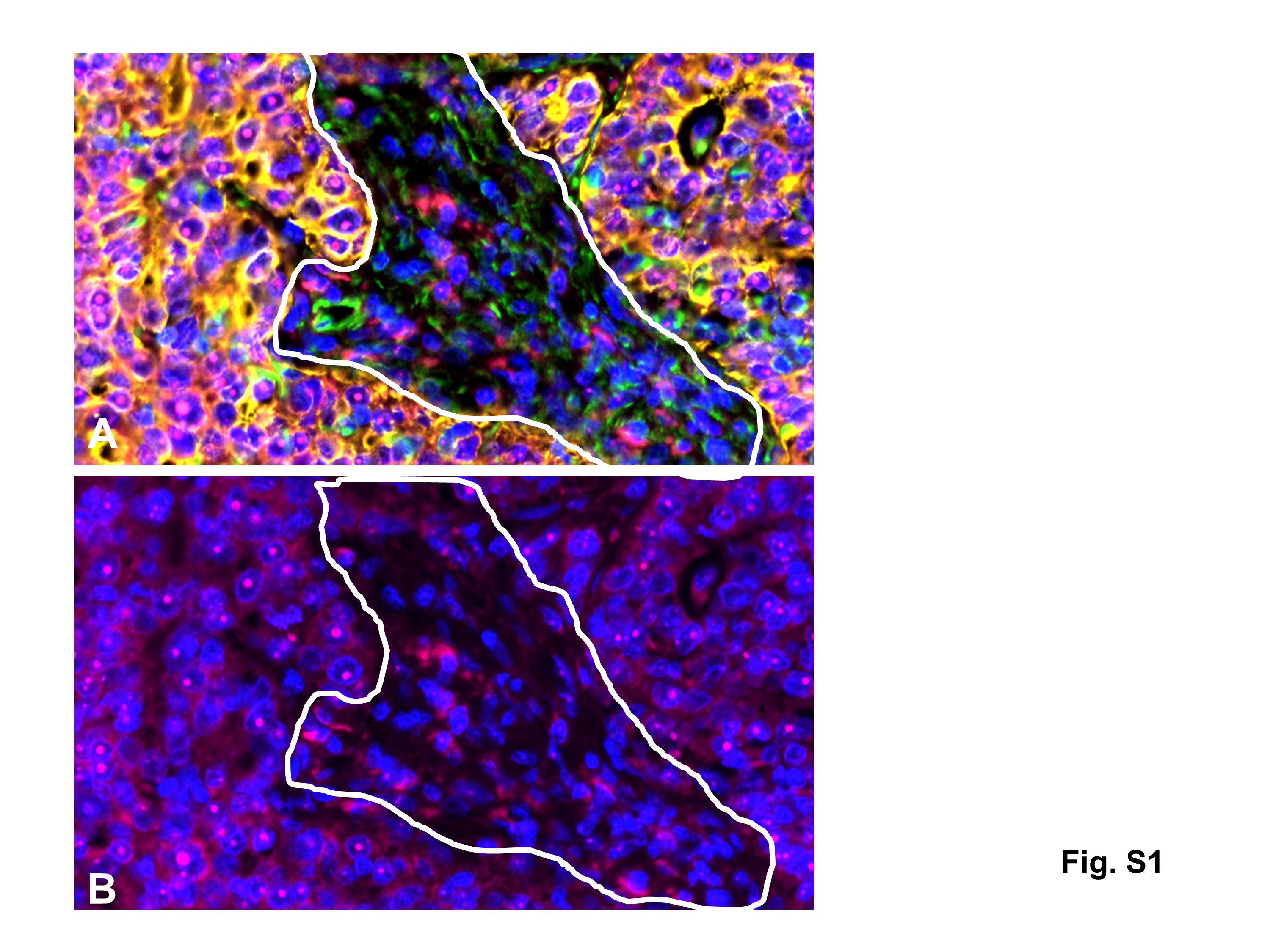

Supplement: S1 Figure — Representative staining of miR-H25 in SEOC patients. In A composited image reporting the nuclei staining (blue), the tumor mask in yellow (cytokeratin), the stromal mask in green (vimentin) and the miR-H25 signal in pink. The region identified in white corresponds to the stromal tissue. In B the same image reporting only the nuclei staining (blue) and the miR-H25 signal (pink). Inside the white region (stromal tissue) the pattern of miR-H25 staining is cytoplasmic while in the epithelial cancer the staining is nuclear. (TIF) [file pone.0114750.s001.tif]

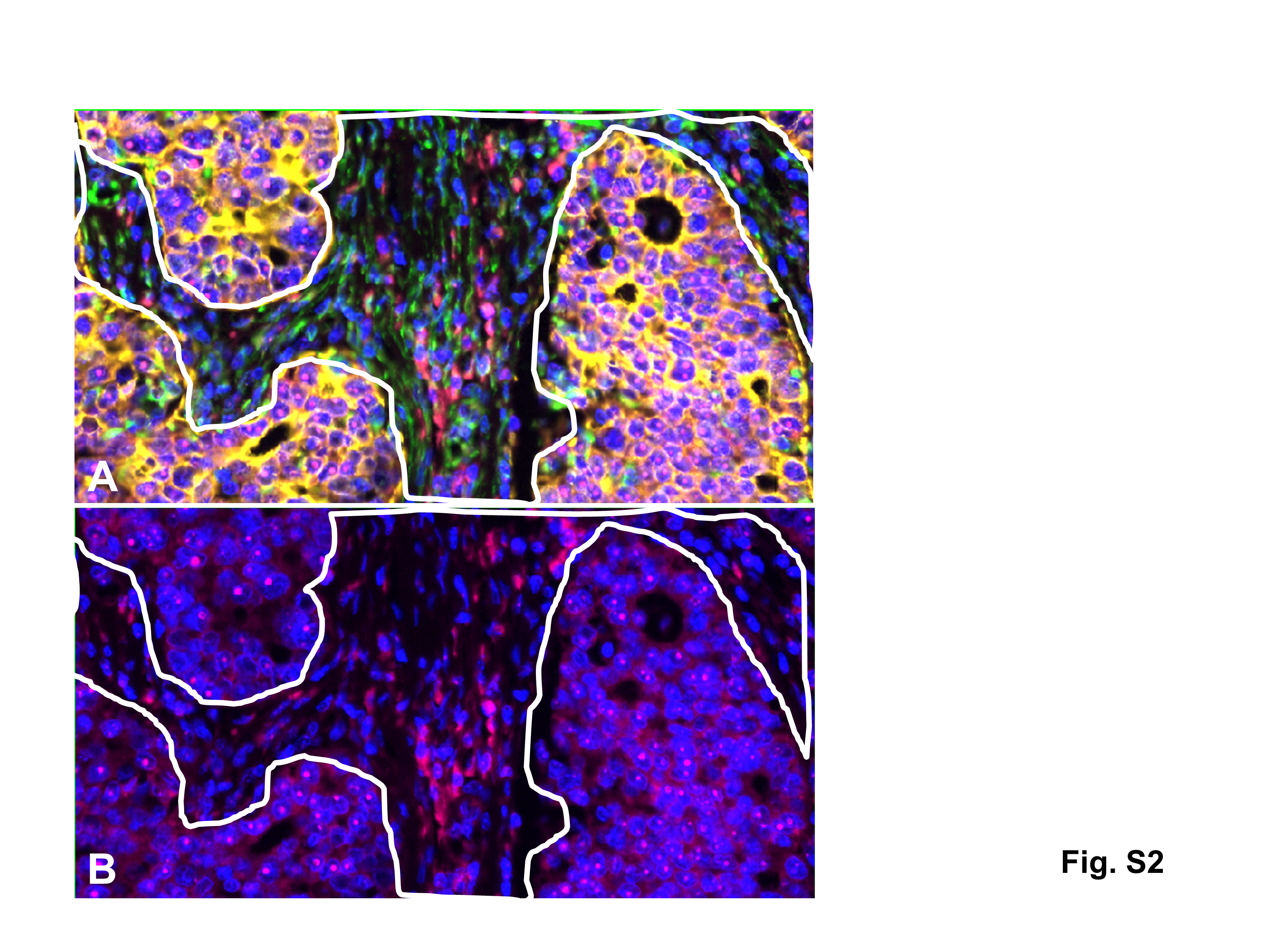

Supplement: S2 Figure — Representative staining of miR-H25 in SEOC patients. In A composited image reporting the nuclei staining (blue), the tumor mask in yellow (cytokeratin), the stromal mask in green (vimentin) and the miR-H25 signal in pink. The region identified in white corresponds to the stromal tissue. In B the same image reporting only the nuclei staining (blue) and the miR-H25 signal (pink). Inside the white region (stromal tissue) the pattern of miR-H25 staining is cytoplasmic while in the epithelial cancer the staining is nuclear. (TIF) [file pone.0114750.s002.tif]

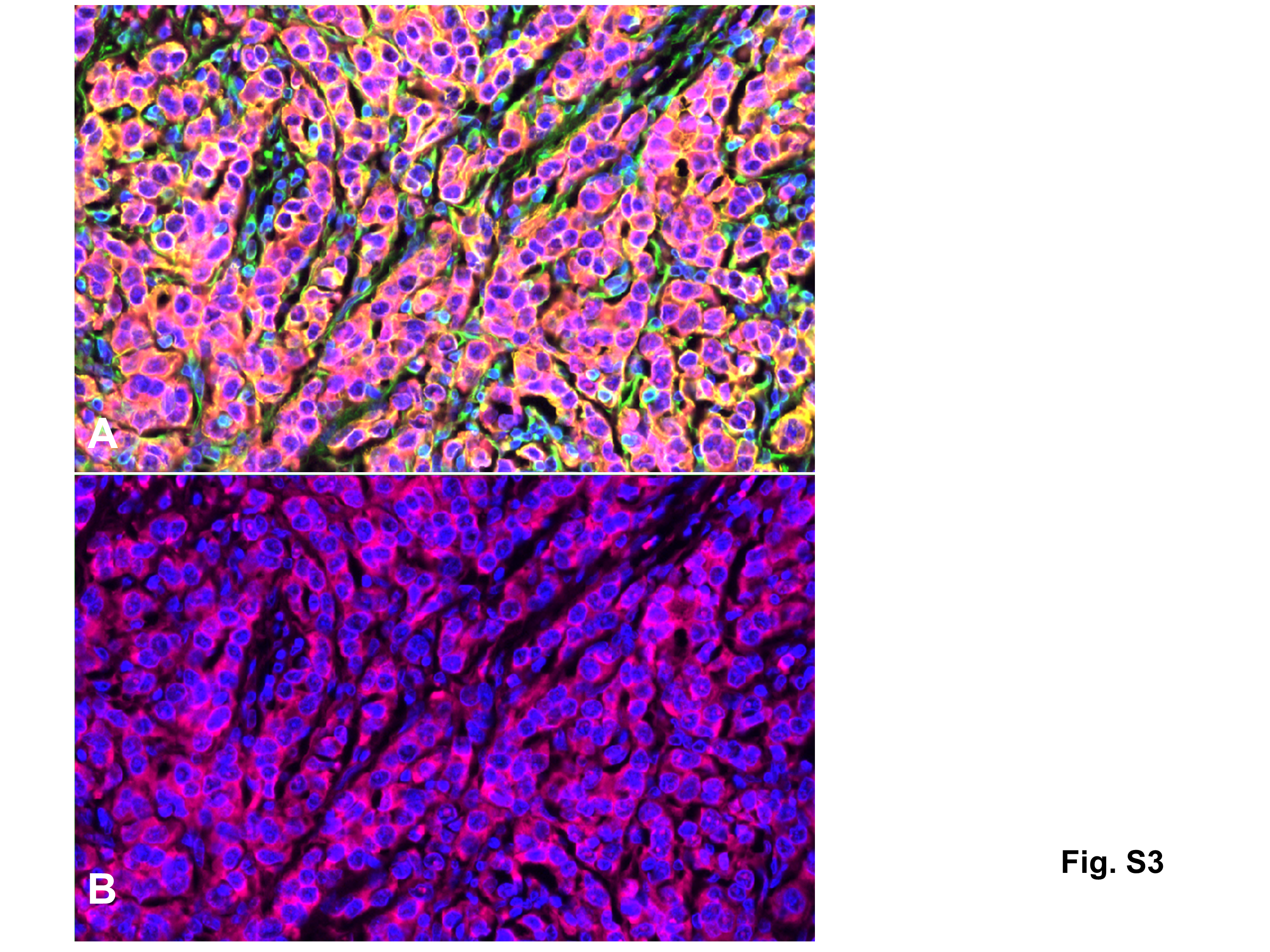

Supplement: S3 Figure — Representative staining of miR-H25 in SEOC patients. In A composited image reporting the nuclei staining (blue), the tumor mask in yellow (cytokeratin), the stromal mask in green (vimentin) and the miR-H25 signal in pink. In B the same image reporting only the nuclei staining (blue) and the miR-H25 signal (pink). In cancer cells it is evident a bright cytoplasmic pattern of staining. (TIF) [file pone.0114750.s003.tif]

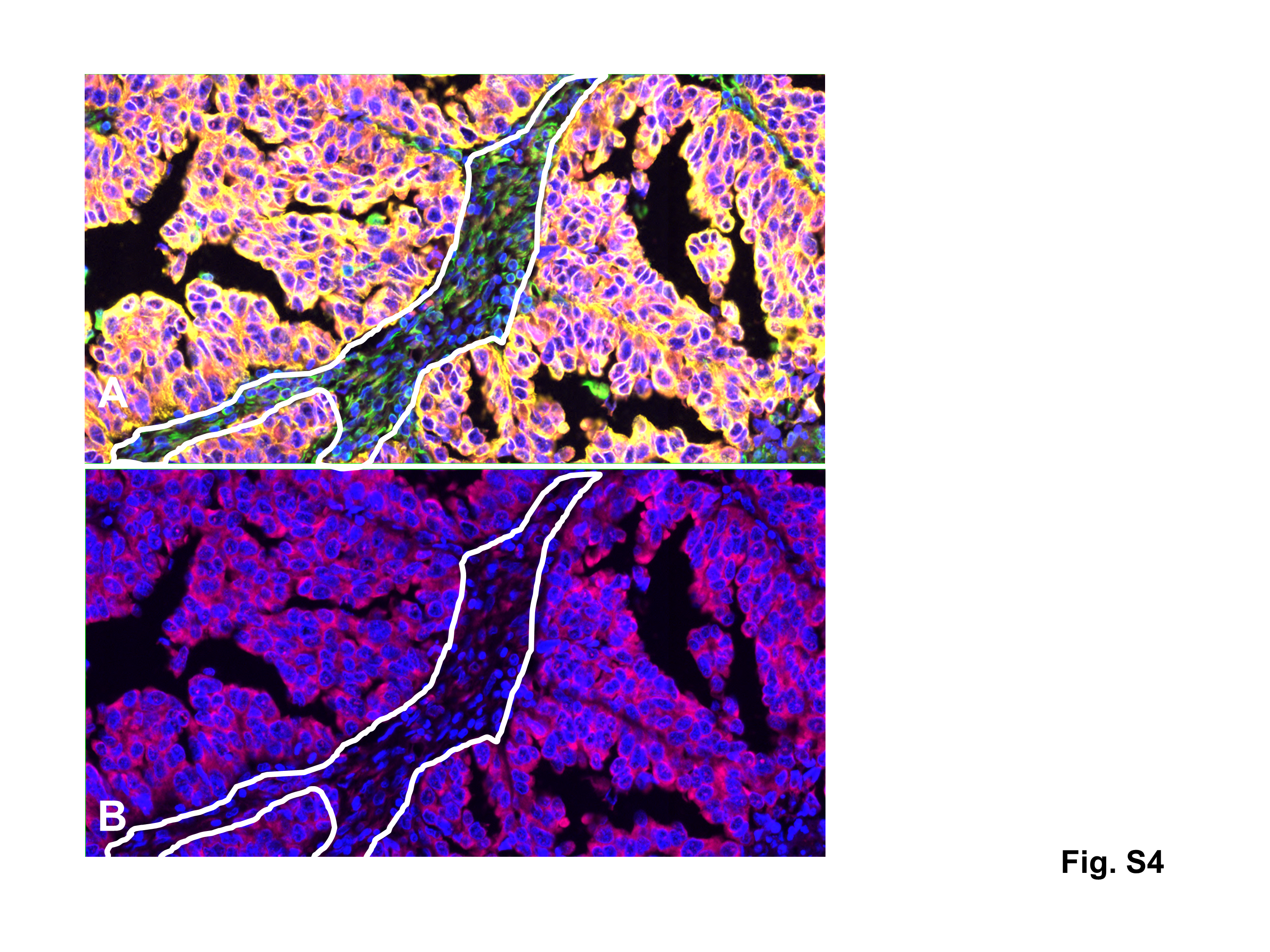

Supplement: S4 Figure — Representative staining of miR-H25 in SEOC patients. In A composited image reporting the nuclei staining (blue), the tumor mask in yellow (cytokeratin), the stromal mask in green (vimentin) and the miR-H25 signal in pink. The region identified in white corresponds to the stromal tissue. In B the same image reporting only the nuclei staining (blue) and the miR-H25 signal (pink). Inside the white region (stromal tissue) the pattern of miR-H25 staining is barely detectable, while in the epithelial cancer the staining is bright with a cytoplasmic pattern. (TIF) [file pone.0114750.s004.tif]

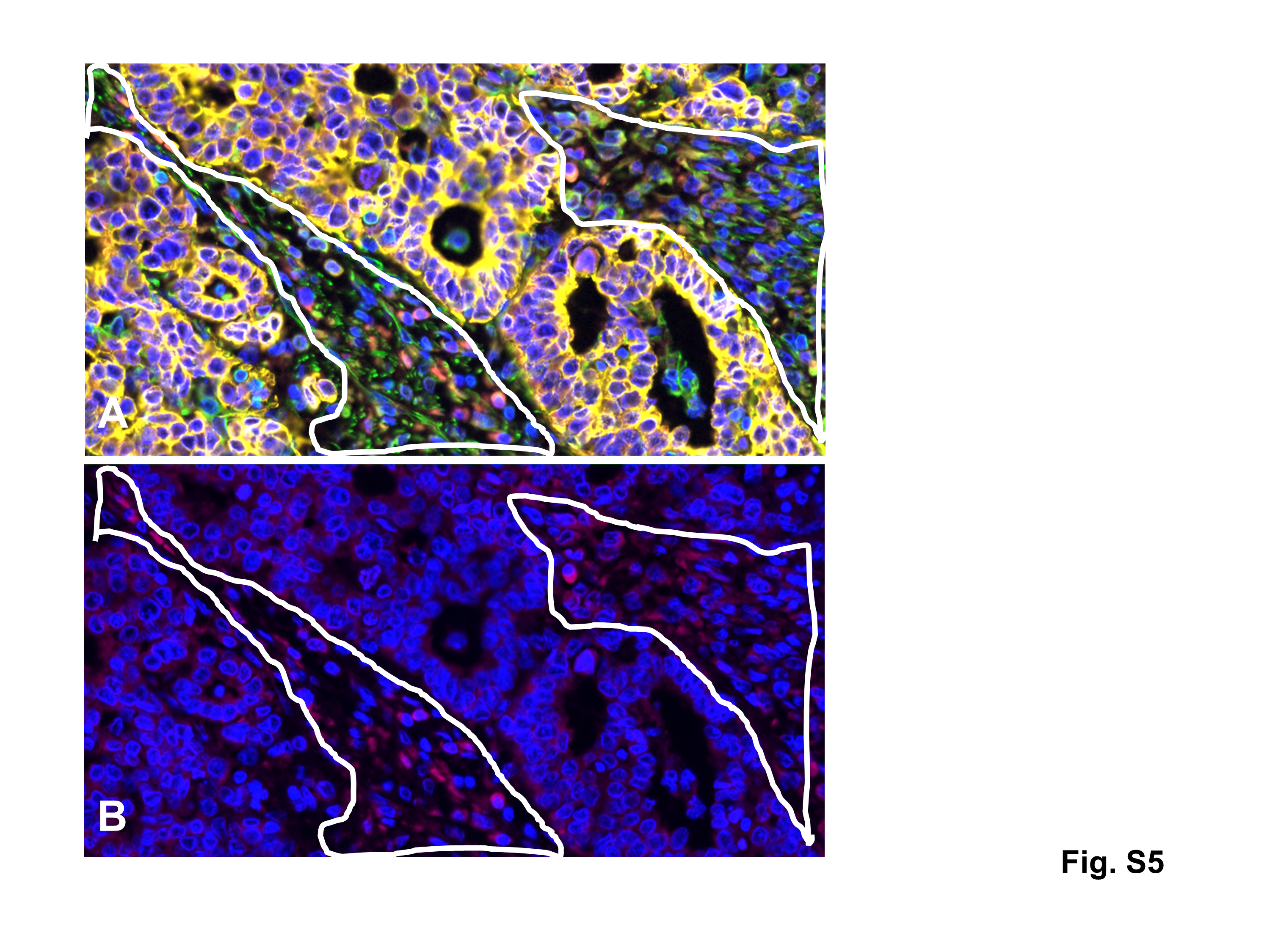

Supplement: S5 Figure — Representative staining of miR-H25 in SEOC patients. In A composited image reporting the nuclei staining (blue), the tumor mask in yellow (cytokeratin), the stromal mask in green (vimentin) and the miR-H25 signal in pink. The region identified in white corresponds to the stromal tissue. In B the same image reporting only the nuclei staining (blue) and the miR-H25 signal (pink). Inside the white region (stromal tissue) the pattern of miR-H25 staining is cytoplasmic while in the epithelial counterpart no miR-H25 is noticeable. (TIF) [file pone.0114750.s005.tif]

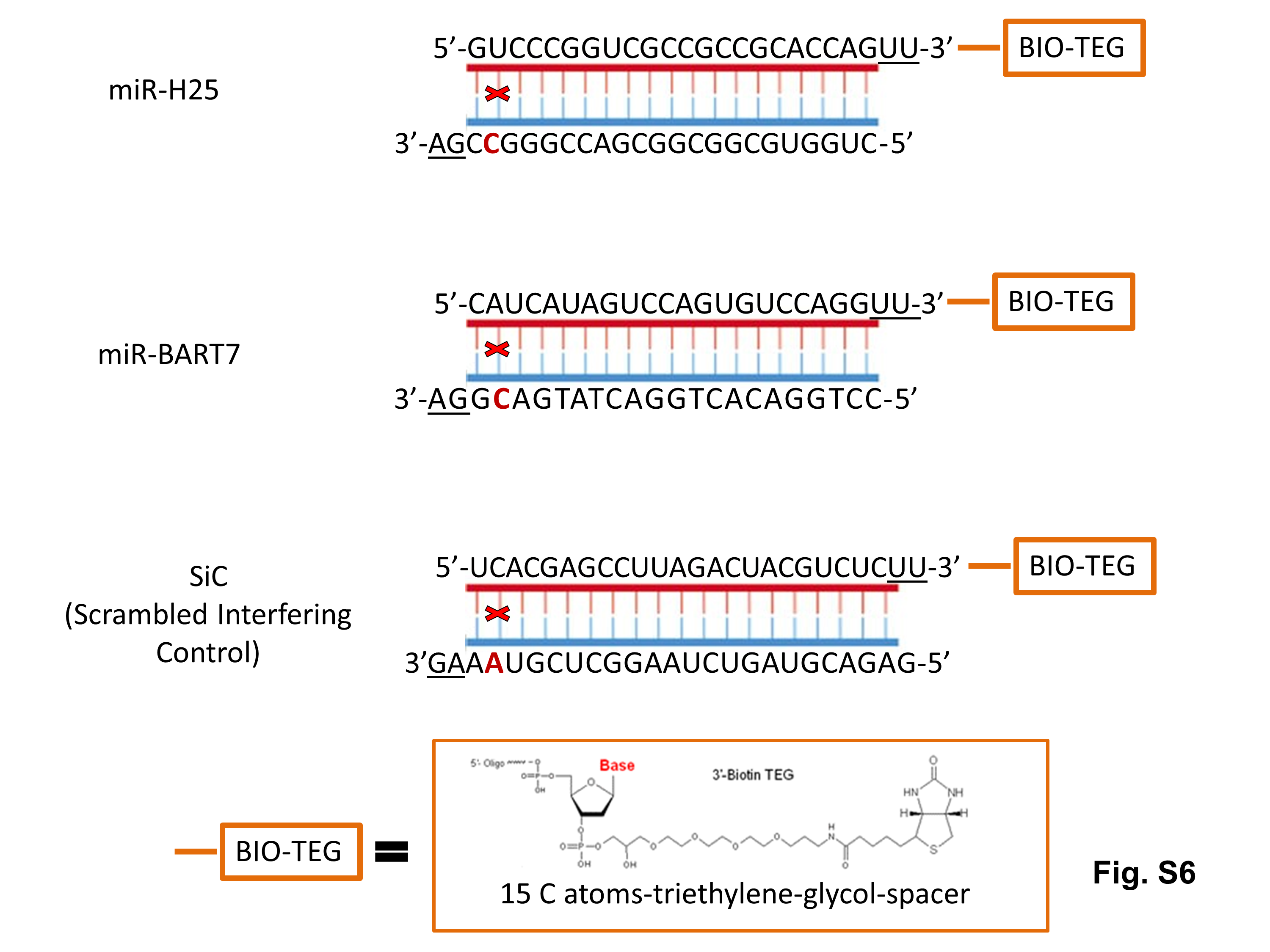

Supplement: S6 Figure — Schematic design of synthetic miR-H25 and miR-BART7. (TIF) [file pone.0114750.s006.tif]
