# Supplementary material for: Herpes Virus MicroRNA Expression and Significance in Serous Ovarian Cancer
Source: PLoS One. 2014 Dec 8;9(12):e114750. doi: 10.1371/journal.pone.0114750 (PMC4259392; doi:10.1371/journal.pone.0114750)
Supplement: S1 Document — Detailed description of the sequencing pipeline. (DOCX) [file pone.0114750.s008.docx]

**High-throughput miRNA sequencing data analysis**

Analysis pipeline was created and divided into three modules.

**Raw data collection module**

Level 1 High-Throughput miRNA sequencing data documenting for 487 Ovarian serous cystadenocarcinoma specimen profiled using Illumina HiSeq sequencer were downloaded from the TCGA data portal to local servers. Permission to access all data was obtained from the Data Access Committee for the National Center for Biotechnology Information Genotypes and Phenotypes Database (dbGAP) at the National Institutes of Health. GeneTorrent shell was installed and used to fetch data on local servers through manifest files, which were created for the study on <https://browser.cghub.ucsc.edu/>. Level 3 gene expression data were also retrieved for 487 ovarian serous cystadenocarcinoma profiled using the U133 Affymetrix chip. High-throughput sequencing data was analyzed according to the pipeline created on CLC genomics workbench 6.5.1 software (CLC GW) and other statistical analysis tools.

**Basic Sequencing analysis module**

Specimen’s raw sequencing data in .bam format (compressed short DNA sequence read alignments format) were first uploaded to the CLC GW software. All specimens’ sequencing data in .bam format were automatically uncompressed and separated into two sequencing files containing mapped and un-mapped read alignment with reference to GRch37-lite. GRCh37-lite is a subset of the full GRCh37 human genome assembly plus the human mitochondrial genome reference sequence from Mitomap.org [1]. Both mapped and un-mapped sequencing alignment files for each specimen were then extracted to obtain sequencing reads. Quality and quantity control was performed to generate reports for each specimen’s mapped and un-mapped total number of reads and average read length.

**Small RNA Sequencing analysis module**

First, all the mapped and un-mapped specimen’s sequencing reads generated through basic sequencing analysis module were trimmed and filtered with maximum read length of 55bp and minimum read length of 15bp. Sampling threshold was set to 1 to not loose even a single unique read. CLC GW’s “extract and count” tool was used for this purpose. As a reference for small RNA sequencing reads’ mapping, three miRNA database (pre-microrna, mature, and GRch37.57 ncRNA) were downloaded [2] [3]. All three databases were then separated into human miRNAs and viral miRNAs. Viral miRNAs from EBV, HSV1, HSV2, KSHV, HCMV, HIV1, HHVB6, JCV, CHV1, and BKV were selected Mapping was performed for each specimen’s mapped and un-mapped small RNA samples through “annotate and merge” tool of CLC GW. This was sub-divided into six different mapping categories shown in Table 1.

| Mapping Category | Reference database |
| --- | --- |
| mapped small RNA samples | human pre-miRNA and ncRNA |
| mapped small RNA samples | human mature RNA and ncRNA |
| mapped small RNA samples | Viral pre-miRNA |
| mapped small RNA samples | Viral mature RNA |
| Un-mapped small RNA samples | Viral pre-miRNA |
| Un-mapped small RNA samples | Viral mature RNA |

No mapping was performed for an un-mapped small RNA samples against human miRNAs on assumption that all human miRNAs reads were annotated to mapped reads when .bam file alignment was performed. Mapping parameters are as shown in Table 2 and Table 3.

**Table 2 Parameters used for mapping small RNA samples against pre-miRNAs database.**

| Additional downstream bases | 4 |
| --- | --- |
| Additional upstream bases | 4 |
| Create annotated sample | Yes |
| Create Mature-grouped sample | Yes |
| Create Precursor/Reference-grouped sample | Yes |
| Maximum mismatches | 2 |
| Missing downstream bases | 3 |
| Missing upstream bases | 3 |
| Strand specific | No |

**Table 3 Parameters used for mapping small RNA samples against mature miRNAs database.**

| Create annotated sample | Yes |
| --- | --- |
| Create Mature-grouped sample | Yes |
| Maximum mismatches | 2 |
| Strand specific | No |

Annotated and counted reads per miRNA per specimen were generated. Summary table was created for all miRNAs in each specimen and then for all miRNAs and all specimen together.

Following the mapping, the small RNAs were classified into the following categories according to where they match: Exact mature 5', Mature 5', Unique exact mature 5', Unique mature 5', Exact mature 3', Mature 3', Unique exact mature '3, Unique mature '3, Exact other, Other, Total.

All mapped miRNAs per specimen were normalized to RPM (reads per million) values with total number of specimen’s sequencing reads. Specimen’s clinical data were then integrated with specimen’s sequencing analysis data.

Reference

1 <http://www.ncbi.nlm.nih.gov/books/NBK49167/>

2 <http://www.mirbase.org/ftp.shtml>

3 [ftp.ensembl.org/pub/release-57/fasta/homo_sapiens/ncrna/Homo_sapiens.GRCh37.57.ncrna.fa.gz](ftp://ftp.ensembl.org/pub/release-57/fasta/homo_sapiens/ncrna/Homo_sapiens.GRCh37.57.ncrna.fa.gz)

Fig. Summary of the pipeline utilized for miRNA-seq of the 487 serous ovarian cancer patients of the TCGA dataset.
